# Impact of social and demographic factors on the spread of the SARS-CoV-2 epidemic in the town of Nice

**DOI:** 10.1186/s12889-023-15917-z

**Published:** 2023-06-06

**Authors:** Eugènia Mariné Barjoan, Amel Chaarana, Julie Festraëts, Carole Géloen, Bernard Prouvost-Keller, Kevin Legueult, Christian Pradier

**Affiliations:** 1 Public Health Department, Université Côte d’Azur, Centre Hospitalier Universitaire de Nice, Route St Antoine de Ginestière. Niveau 1, CS23079, Nice cedex 3, 06202 France; 2 Université Côte d’Azur, UR2CA, Nice, France

**Keywords:** COVID-19, Incidence, Social inequalities, Risk factors

## Abstract

**Introduction:**

Socio-demographic factors are known to influence epidemic dynamics. The town of Nice, France, displays major socio-economic inequalities, according to the National Institute of Statistics and Economic Studies (INSEE), 10% of the population is considered to live below the poverty threshold*, i.e.* 60% of the median standard of living.

**Objective:**

To identify socio-economic factors related to the incidence of SARS-CoV-2 in Nice, France.

**Methods:**

The study included residents of Nice with a first positive SARS-CoV-2 test (January 4-February 14, 2021). Laboratory data were provided by the National information system for Coronavirus Disease (COVID-19) screening (SIDEP) and socio-economic data were obtained from INSEE. Each case’s address was allocated to a census block to which we assigned a social deprivation index (French Deprivation index, FDep) divided into 5 categories. For each category, we computed the incidence rate per age and per week and its mean weekly variation. A standardized incidence ratio (SIR) was calculated to investigate a potential excess of cases in the most deprived population category (FDep5), compared to the other categories. Pearson’s correlation coefficient was computed and a Generalized Linear Model (GLM) applied to analyse the number of cases and socio-economic variables per census blocks.

**Results:**

We included 10,078 cases. The highest incidence rate was observed in the most socially deprived category (4001/100,000 inhabitants *vs* 2782/100,000 inhabitants for the other categories of FDep). The number of observed cases in the most social deprivated category (FDep5: *N* = 2019) was significantly higher than in the others (*N* = 1384); SIR = 1.46 [95% CI:1.40–1.52; *p* < 0.001]. Socio-economic variables related to poor housing, harsh working conditions and low income were correlated with the new cases of SARS-CoV-2.

**Conclusion:**

Social deprivation was correlated with a higher incidence of SARS-CoV-2 during the 2021 epidemic in Nice. Local surveillance of epidemics provides complementary data to national and regional surveillance. Mapping socio-economic vulnerability indicators at the census block level and correlating these with incidence could prove highly useful to guide political decisions in public health.

**Supplementary Information:**

The online version contains supplementary material available at 10.1186/s12889-023-15917-z.

## Introduction

Social measures intended to contain the SARS-CoV-2 epidemic were applied in France according to indicators published by the French Public Health Agency *Santé Publique France* (SPF): incidence rate, hospitalisation rate and positivity rate (PCR or antigenic test). During the fourth quarter of 2020 there was a surge in the COVID-19 epidemic in France. Consequently, the government initially ordered a nationwide lockdown on October 29, 2020, which was lifted on December 15, 2020 while maintaining a country-wide curfew between 8 pm and 6 am for the end-of-year celebrations.

Nice is situated in the Alpes-Maritimes department in South-eastern France where, during the week starting on November 16, 2020, the incidence rate for SARS-CoV-2 per 100,000 inhabitants exceeded the mean rate recorded at the national level (178/100,000 inhabitants versus 166/100,000 inhabitants) [1, 2]. On December 18, 2020 it crossed the national alert threshold of 250/100,000 inhabitants set by the government. Thus, on January 2, 2021, as in 14 other French departments a tightening of the curfew from 6 pm to 6 am was announced on the radio by the Health minister.

Despite these measures aimed at controlling the epidemic, the SARS-CoV-2 incidence rate in the Alpes Maritimes department was the highest in metropolitan France (continental France and Corsica) at the start of 2021 and progressed between the first to the sixth week from 456 to 577 per 100,000 inhabitants, *i.e.* a continuously increasing rate which remained significantly higher than the government’s national alert threshold [3, 4].

Social and economic factors have always played a major role in epidemic dynamics [5] even in affluent countries [6, 7]. Several studies have focused on their association with mortality and progression of the SARS-CoV-2 epidemic in Europe and worldwide. In a study of 12 European countrys, Amdaoud et al. found that the share of older people in the population, GDP per capita, distance from achieving EU objectives, and the unemployment rate are correlated with high COVID-19 mortality [8–12]. In the municipalities of Santiago, Chile, there was a strong association between socioeconomic status and mortality, measured by either COVID-19, attributed deaths or excess deaths [13].

In Barcelona, Spain, the incidence of COVID-19 disease was higher in some poor neighborhoods and the risk ratio (RR) increased in the poorest groups compared to the richest ones [14–17]. Recently, results of the first study on social inequality in France and COVID-19 diagnosis highlighted the major role of social deprivation [18].

Each year, the French National Institute of Statistics and Economic Studies (*Institut national de la statistique et des études économiques: INSEE*) publishes socio-economic data grouped by census blocks (Ilots Regroupés pour l’Information Statistique: IRIS). A census block is the smallest geographical unit for which INSEE data are available and cover relatively similar geographic areas with regard to socio-economic characteristics. The socio-economic data includes counts of population, households or residences at the census block level, classified according to social, economic and demographic characteristics, *i.e.,* among other variables, employment, income, housing, level of education or family structure [19].

The geographical boundaries for the census blocks were obtained from the National Geographic Institute. In 2017, the most recent year for which socio-economic data were available per age group and per census block, the population of the Alpes- Maritimes department numbered 1,083,310, among whom 340,017 lived in the town of Nice which includes 146 census blocks, with between 1,142 and 4,442 inhabitants (mean 2,328 inhabitants); two census blocks include the central railway station and the airport.

The town of Nice displayed major socio-economic inequalities [20]. The poverty threshold is defined by the INSEE as 60% of the median available income per household. The poverty rate is the proportion of the population living below this threshold within each census block. In Nice, there are 14 census blocks with > 35% of the population living below this threshold; this concerns 34,358 inhabitants (10% of the town's population). The published socio-economic indicators were complete for 144 of the Nice census blocks.

Based on these data, the Surveillance centre for medical causes of death (*Centre d'épidémiologie sur les causes médicales de Décès: CépiDc*), which conducts statistical analyses of deaths in France, has developed a composite indicator of social disadvantage, i.e. the « French Deprivation index» (FDep) [21] which provides a synthetic view of social inequalities. To our knowledge, the association between socio-economic characteristics and the incidence of SARS-CoV-2 in a large town at the grouped census block level according to an index of social disadvantage has not been studied in France. We calculated the incidence rate of SARS-CoV-2 in Nice during the first 6 weeks of 2021 to measure the association between the progression of the epidemic and the various factors related to social inequality and to identify at risk population clusters.

## Methods

The study included residents of Nice with a first positive SARS-CoV-2 PCR or antigenic test result obtained between January 4, 2021 and February 14, 2021. Data were provided by the National information system for COVID 19 screening (*Système d’information national de suivi du dépistage de la COVID-19: SI-DEP*) which has been recording all positive SARS-CoV-2 tests in France since May 2020. Variables of interest included age, date of first positive test and residence coordinates. We considered a first positive test to be the one from a patient who had not tested positive during the previous 30 days.

Quality control consisted in eliminating duplicates, correcting inconsistent data, and completing missing addresses. The addresses of cases living in collective accommodation (nursing homes or long-term accommodation) were identified. Incomplete addresses were corrected thanks to the partial information previously retrieved from each case. Each address was entered in a Geographic Information System (ARCGIS 10®) to allocate it to a census block. All GIS techniques and map layouts were performed using ArcMap v.10.5 (ESRI, Redlands, CA, USA). We excluded the blocks containing the airport and the railway station as these are considered uninhabited, and in which data for certain socio-economic indicators were lacking. The socio-economic data were obtained from the 2017 national census. For each of the 144 census blocks constituting the town of Nice, we computed the social deprivation index (FDep) based on four socio-economic variables: i) the median annual income per consumption unit in the household, ii) the percentage of workers in the active population, iii) the percentage of graduates in the population over 14 years of age and, iv) the unemployment rate (Additional file 1). This index allowed us to create a qualitative variable, using Jenks’ natural breaks classification method, which optimizes the arrangement of a set of values into « natural» classes. A class range is composed of items with similar characteristics [22]. We thus divided the FDep index into 5 classes, ranging from the least deprived (lowest scores) to the most deprived (highest scores), with the following score ranges: FDep1[-2.84/-1.36], FDep2[-1.35/-0.48], FDep3[-0.47/0.21], FDep4[0.22/0.91], FDep5[0.92/1.83] (Additional file 2). For each FDep category, we calculated the SARS-CoV-2 incidence rate per age group, per week and for the overall study period. We also calculated the mean weekly variation (MWV) in incidence rate as follows: we modelled the logarithm of the crude weekly rates over the study period [t1-tx] [23].

$$\mathrm{Ln} \left(\mathrm{incidence\,rate}\right) =\mathrm{ax}+\mathrm{b}$$

$$\mathrm{a}=\mathrm{estimated\,slope\,of\,the\,regression\,line}$$

$$\mathrm{x}=\mathrm{N\,weeks}$$

$$\mathrm{b}=\mathrm{baseline\,weekly\,incidence\,rate\,at\,the\,start\,of\,the\,study}$$

$$MWV={100}^{*}\left({\mathrm{e}}^{\mathrm{a}}-1\right)$$

Changes in rates are expressed as percentages with regard to the previous week. The differences were considered statistically significant for a *p*-value < 0.05.

A standardized incidence ratio (SIR) with a 95% confidence interval was calculated to investigate an excess number of cases of SARS-CoV2 in the most deprived population (FDep5), compared to the reference population (FDep categories 1,2,3,4) which, according to our assumption, has a lower incidence rate [24].

$$SIR = \frac{O}{E}$$

O: number of observed cases in FDep5

E: number of « expected » cases by applying the age-adjusted morbidity rate among the reference population (FDep1 to FDep4).

We then calculated the correlation between the number of new cases per census block and each of the socio-economic variables within these blocks (Pearson’s correlation coefficient). Next, we used a GLM model to study the link between the dependent variable (number of cases) and the independent variables we selected based on Ridge’s method [25], which reduces the effect of colinearity, *i.e.* the correlation between independent variables. This led us to remove the median income variable.Lastly, the non-parametric Kruskall-Wallis test was used to analyse age. Statistical analyses were performed using SAS® and SPSS® software packages.

## Results

### Incidence

**Fig. 1:**
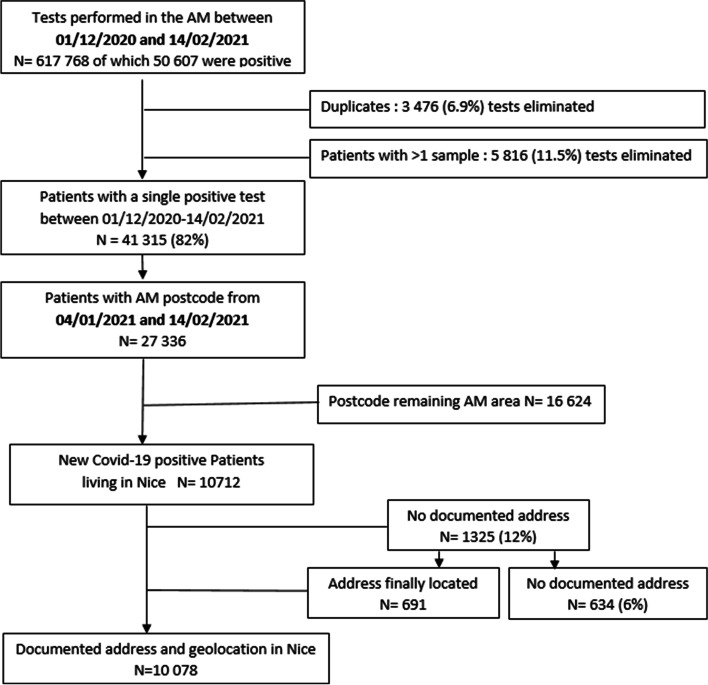
Flow chart

During the first 6 weeks of 2021, 27,336 individuals with a known zip code in the Alpes-Maritimes department had a positive SARS-CoV-2 PCR test result. Among these, 10,712 (39%) were living in Nice, and 10,078 of them had an identified address: 1325 (12%) lacked an address allowing geolocation (missing number, collective accommodation lacking an address, missing addresses). Following further verification, the number of missing addresses was reduced to 634 (6%) (Fig. 1) as we were able to complete the partial information previously obtained for each case.

**Fig. 2:**
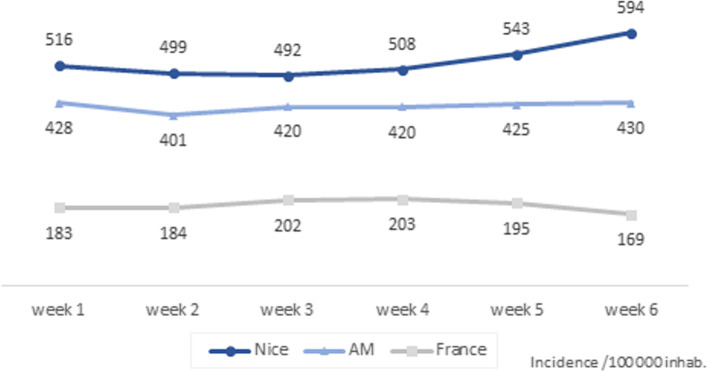
Weekly SARS-CoV-2 incidence rate in Nice, Alpes-Maritimes and France during the study period

Over this period, the town of Nice was an area of very high transmission of SARS-CoV-2, which markedly progressed from week five onwards (February 1, 2021 to February 7, 2021) (Fig. 2, Additional file 2). During the first three weeks of January, 4762 cases (47.3%) were diagnosed, and 5316 (52.7%) were identified over the three following weeks.

There was a significant drop in the median age of cases between the first and the sixth week (44[IQR: 27–63] years versus 39[IQR: 24–55] years; *p* < 0,001). During the first four weeks, positive tests concerned patients above 80 years of age while this proportion dropped significantly during the last two weeks (10.3% vs 6.7%; *p* < 0.001). We also computed the incidence rate excluding those living in collective accommodation, with similar results (Additional file 3).

### Incidence rate according to the social deprivation index

**Fig. 3:**
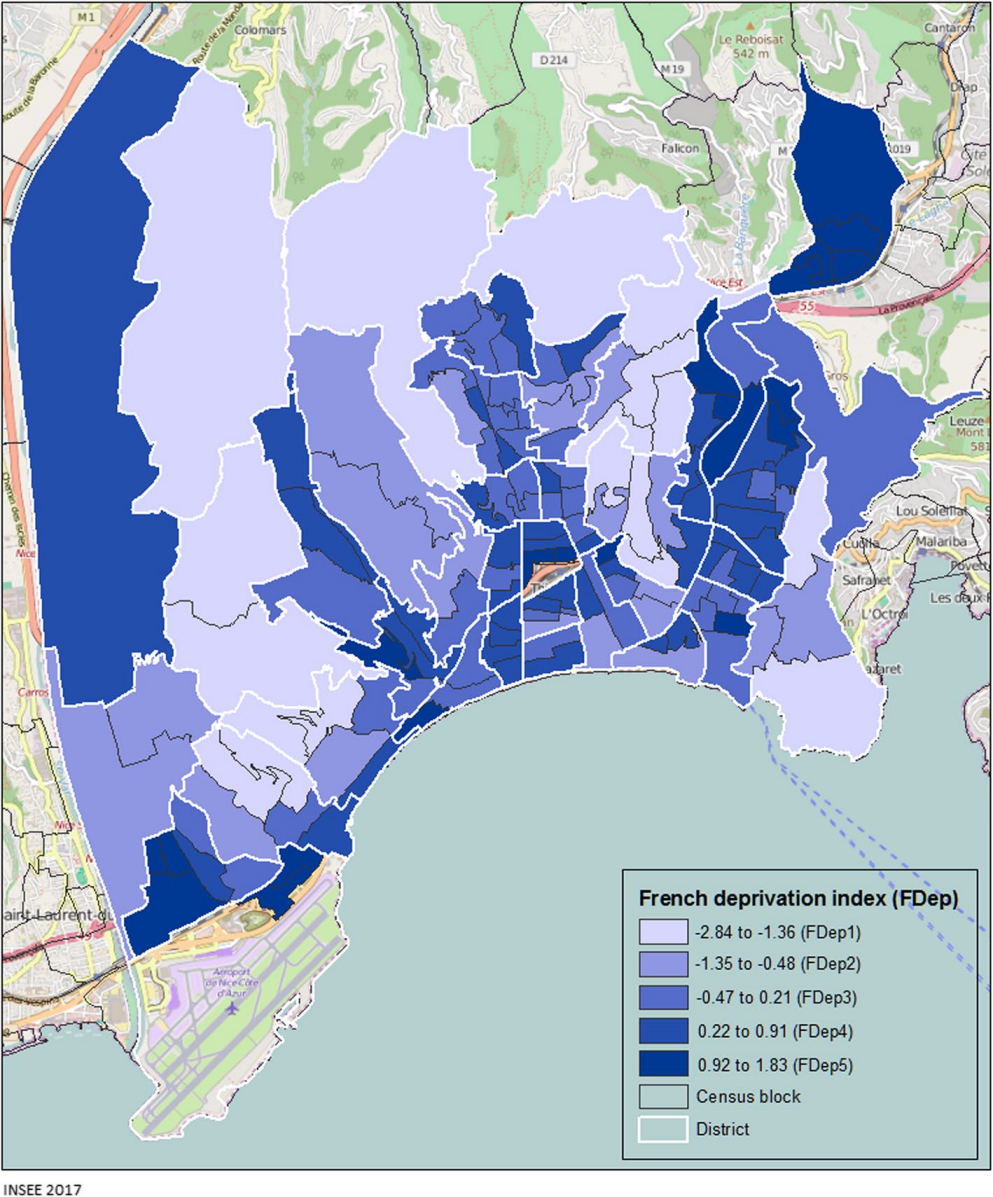
Distribution of the social deprivation index (FDep) across the town of Nice

Figure 3 shows the distribution of the FDep index in the town of Nice. Darker colours show the most deprived census blocks (FDep5). Twenty-one census blocks (15% of the 144) in the FDep5 category were identified, harbouring 50,468 inhabitants (15% of the Nice population).

The FDep distribution per quintile is shown in Additional file 4.

The incidence rate, number of cases and their frequency for each FDep category are shown in Table 1. The incidence rate over the study period among the most deprived category (FDep5) reached 4,001/100,000 inhabitants, *i.e*. higher than that observed among the remaining population of Nice: 2,782/100,000 habitants. Likewise, the number of observed cases in the FDep5 category (*N* = 2019) (worst level of social deprivation) was significantly higher than expected (*N* = 1384) SIR = 1.46 [95% CI: 1.40–1.52; *p* < 0.001]. Over the 6-week study period, the incidence rate also increased significantly among residents within the FDep5 category, *i.e.* + 1.6% [95%CI: 1.1–2.3; *p* = 0.002] and to a lesser extent in the FDep3 category: + 1.2% [95%CI: -0.2- 2.5; *p* = 0.05].

**Table 1:** Trends in incidence rate for the study period according to social deprivation category

	Number of census blocks	Population	Number of cases	Incidence rate#	Mean variation in weekly incidence rate	95% CI	*p*
FDep1	18	45 622	1 136	2 490	1.7	[-1.1 ; 4.7]	0.1
FDep2	20	45 925	1 323	2 881	1.2	[-1.7 ; 4.2]	0.3
FDep3	42	91 329	2 527	2 767	1.2	[-0.2 ; 2.5]	0.05
FDep4	43	106 092	3 054	2 879	1.5	[-1.7 ; 4.8]	0.2
FDep5	21	50 468	2 019	4 001	1.6	[1.1 ; 2.3]	0.002

^#^ per 100 000 inhabitants and from 2021/01/04 to 2021

**Fig. 4:**
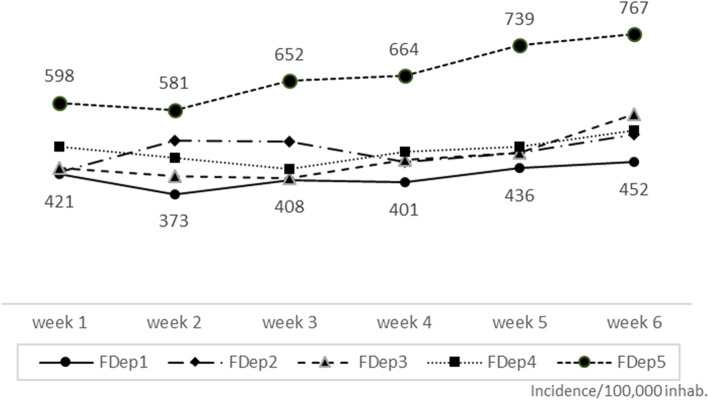
Weekly SARS-CoV-2 incidence rate in Nice according to social deprivation category

The result obtained using the Townsend index for classes is similar to that found with the FDep index (Additional file 5). Over the 6 weeks during which the study was conducted, the incidence rate among the most deprived category was consistently higher than among the remaining categories (Fig. 4).

**Fig. 5:**
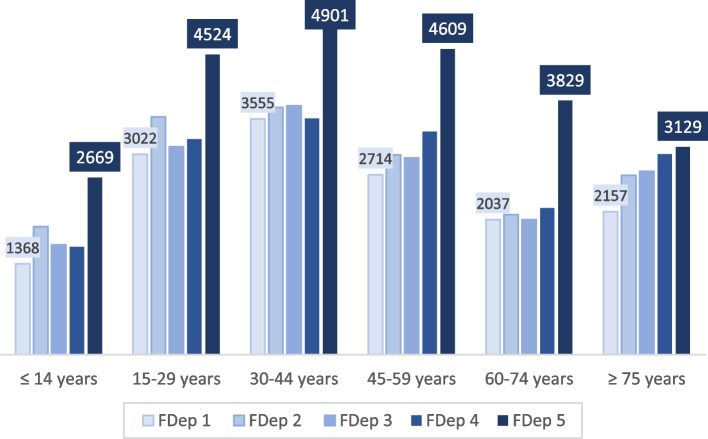
Incidence rate over the study period per age group according to social deprivation category

Figure 5 shows the incidence rate per age group and for each social deprivation category. The highest incidence rate was observed in the most socially deprived areas (FDep5) for each age group (from 2669 to 4901 cases /100,000 inhabitants) and the lowest among those below 14 years of age (1368 to 2669 cases /100,000 inhabitants). Conversely, the incidence rate among persons above 75 years of age living in the most deprived census blocks did not increase as much as among the remaining population (FDep 1 to 4) (Additional file 6).

### Correlation between number of cases and socio-economic indicators

Details of the number of cases according to socio-economic indicators per census block are shown in Table 2, according to Pearson’s correlation coefficient as well as according to the GLM model (Additional file 7). Both tests showed a statistically significant association between the number of cases and poverty indicators: (poverty rate, share of taxable households, people benefiting from state-provided free full health insurance, single-parent families), type of professional activity (farmers, intermediate professionals which include healthcare workers, and employees), and educational level (primary school certificate). Pearson’s correlation coefficient also identified overcrowded main residences, rented or owned apartments as main residence, craftsmen, workers and unemployed. Using the GLM model we also found an association with population density as well as with stated median income (Additional file 8).

**Table 2:** Pearson correlation and Generalized linear model

	Pearson's correlation		Generalized linear model	
	R	*p*-value	*p*-value	CI 95%
Population density	0.04585	0.5826	0.0163^*^	[-0.001 ; -0.0001]
Overcrowded main residence	0.47047	< .0001^*^	0.6317	[-0.1136 ; 0.1871]
House as main residence	0.08402	0.3133	0.1125	[-0.3122 ; 0.0328]
Appartment as main residence	0.33198	< .0001^*^	0.1822	[-0.2741 ; 0.0521]
Owners of main residence	0.04065	0.6261	0.2994	[-0.0808 ; 0.2627]
Tenants of main residence	0.48458	< .0001^*^	0.2606	[-0.0683 ; 0.2524]
Single-parent families	0.61178	< .0001^*^	0.0092^*^	[-0.3422 ; -0.0484]
Poverty rate	0.20816	0.0162^*^	0.0120^*^	[0.4640 ; 3.7593]
Median income (€)	-0.29505	0.0003^*^	-	-
Taxable households	-0.27553	0.0009^*^	0.0301^*^	[0.1459 ; 2.8772]
Farmers	0.20520	0.0130^*^	0.0004^*^	[1.4730 ; 5.0971]
Artisans, shopkeepers, company managers	0.17366	0.0361^*^	0.1147	[-0.3505 ; 0.0379]
Executives, Higher professions	-0.07670	0.3575	0.2555	[-0.0352 ; 0.1324]
Intermediate professions	0.24280	0.0031^*^	0.0337^*^	[0.0066 ; 0.1645]
Employees	0.54404	< .0001^*^	0.0002^*^	[0.0798 ; 0.2568]
Workers	0.65369	< .0001^*^	0.6952	[-0.1610 ; 0.1074]
Unemployed	0.52363	< .0001^*^	0.4262	[-0.1560 ; 0.0659]
Population > 14 years with no diploma nor Primary school certificate	0.66709	< .0001^*^	0.0022^*^	[0.0243 ; 0.1110]
Population with full state-provided health insurance	0.38281	< .0001^*^	0.0237^*^	[0.0113 ; 0.1584]

* statistically significant

## Discussion

This study was conducted at a time when the town of Nice had one of the highest SARS-CoV-2 incidence rates in France. It shows that the incidence rate was significantly higher in the most deprived census blocks regardless of the age group. We also report a more rapid weekly progression among the most deprived population (FDep5). Lastly, we observed a decrease in the number of cases in the over-80 age group, starting in the 5th week of 2021, which could reflect an early effect of the administration of the vaccine that was initiated in this population at the end of December 2020.

In the GLM model, a statistically significant association was found between the number of cases and the various socio-economic indicators that characterize the most disadvantaged populations, such as population density, poverty rate, single parent families, taxable households, farmers, intermediate professions, employees, primary school education and people benefiting from free full health insurance coverage. These results are in line with studies showing a correlation between population density (Additional file 9) and the spread of the epidemic [26–29], and with European studies highlighting an increase in the number of SARS-CoV-2 cases among low-income earners [9, 16, 30].

In Pearson’s correlation analysis, we also showed the living environment to be an important determinant of viral spread: census blocks most affected by the virus were those where cases lived in apartments and overcrowded dwellings. This has also been reported in a study on SARS-CoV-2 seroprevalence on a representative sample of the French population [31]. The major role of the urban model and housing type had already been reported for other infectious diseases [7].

The number of cases was also found to be associated with the type of professional activity, with more cases among craftsmen, workers and the unemployed according to Pearson’s correlation. These were not identified by the GLM model. For both types of analysis, farmers, intermediate professions and employees had higher incidence rates. Artisans, shopkeepers, company managers, executives and professions requiring a higher level of education were shown to have lower incidence rates. The employees category includes most « essential» professions, i.e. people who could not work remotely and who were therefore in direct, unavoidable, and repeated contact with the rest of the population [14, 32, 33]. These jobs are more frequent among residents of the most deprived census blocks and may have thus favoured the spread of SARS-CoV-2 in these areas [14]. Farming was also a profession associated with the number of cases; in Nice, there are very few and they mainly reside in one of the deprived census blocks. There was correlation with case numbers according to the GLM model for so-called intermediate professions which include non-medical health professionals [34]. A study conducted in New York in March 2020, and another in the United States as a whole, also found a link between the type of occupation and the risk of transmission [35, 36].

Our results on the incidence rate of SARS CoV-2 in deprived areas are also in line with the study on COVID-19-related mortality conducted at the start of the epidemic by the Health office for the Ile de France region (Office Régionalde Santé Ile-de-France), in which excess mortality was closely linked to the social and urban geography of the population [37].

A higher degree of social deprivation would thus contribute to the increase in transmission related to overcrowded living conditions, the cohabitation of generations in the same dwelling and the greater frequency of housing in apartments, particularly when crowded. Limited possibilities of working remotely, inherent in the type of profession of this section of the population, would also be among the factors favouring transmission [23]. By forcing people living in high-incidence areas such as the town of Nice to remain at home from 6:00 pm to 6:00 am, the curfew may have contributed to a greater risk of SARS-CoV-2 transmission among such most deprived populations [25]: employees with critical jobs involving frequent contacts could introduce the virus into their households. Besides, because of de curfew, members of the general population would gather in numbers at similar hours in crowded essential public areas such as supermarkets.

Complying with hygiene measures, conforming to a strict lockdown or isolating oneself in an apartment without outdoor areas is undoubtedly more complicated than in a house with a garden, the more so when it comes to over-crowded apartments or limited surfaces. Our results could warrant surveillance at a smaller scale than a department, even within municipalities, to accurately chart the progression of COVID-19 or any other epidemic. This could thus lead to the rapid implementation of intervention strategies targeting the areas that need them most.

The methodology chosen for this study may have induced certain limitations regarding our results.

The SIDEP database which we used offers the advantage of providing all cases recorded in a given geographical area. In a similar study, focusing on France as a whole, for which geolocation was essential, the authors also reported a missing address in 20.5% of cases. To compensate for these missing data, they chose to allocate cases to census blocks on a probability basis [18]. Although it was possible to reduce the number of missing addresses, our results may be biased as we were unable to take these cases into account in our analysis.

Although the study was conducted in 2021, we chose to use the 2017 INSEE data, which was the most recent year for which distribution per age group and census block was available, as well as the related socio-economic data. The delay between the study period and the one for which socio-economic data are available has already been mentioned in other French studies [18]. Between 2017 and 2019, the latest year for which the overall population in Nice is known, the number of inhabitants increased by 1.6%, from 340 017 to 345 528. We thus considered that this bias did not compromise the validity of our results, despite potential minor changes in socio-economic data over the 4-year period.

In our view, the FDep was the most suitable social deprivation index for our population and provided the most complete results for the town of Nice. In contrast to the Townsend index developed for the United Kingdom population in 1987 [38] (Additional file 5), the FDep index was specifically developed and validated for the French population. The Townsend index was the first index allowing to identify socially deprived populations, but in our view, it has somewhat lost its relevance especially in a town, as is the case in our study. Car use in the city is increasingly controversial and not owning a car does not necessarily reflect social deprivation. As for the Ecological Deprivation Index (EDI), developed for the French and European population, it included 10 weighted variables [39], while the INSEE data per census block in Nice were not complete for all these 10 variables. The FDep, with its 4 variables, could be computed almost completely for the whole of Nice. We used Jenk’s method to create a categorical variable.Indeed, this method does not classify data in an arbitrary fashion, subdividing the population in groups of 20% as with quintiles, but according to their uniformity, with thresholds that we feel are more in line with reality. When applied to a transmissible disease, the Jenks algorithm can limit within-class variance while maximising variance between classes [17].

The missing data for certain variables, such as the poverty index in 5 of the 144 studied census blocks, could have biased our correlation results. However, since two of these census blocks were in the most deprived category, two in the most affluent category, and one in the middle category, we considered that these missing data had little impact on our results.

Until individual specificities are taken into account, the FDep index, which is a simple composite index, can provide the basis for a synthetic approach at the level of a particular territory. Considering that the socio-economic status is a multidimensional and complex concept, and with Khalatbari-Soltani [40], we believe that surveillance of infectious diseases should take socio-economic data into account. This would allow precise identification of at-risk groups in order to implement targeted and equitable public health policies [31].

## Conclusion

This descriptive study of the SARS-CoV-2 epidemic according to socio-economic variables, conducted in the town of Nice over six weeks in 2021, shows that people living in the most deprived areas of a large town were those most impacted by the COVID-19 epidemic and among whom the epidemic spread fastest. As for other infectious or non-infectious conditions, social inequalities should be considered to ensure comprehensive and targeted interventions for future epidemics. Local surveillance of epidemics provides complementary data to national and regional surveillance. Mapping socio-economic vulnerability indicators such as that recorded by the INSEE at the census block level and correlating these with incidence rates could prove highly useful to guide political decisions in public health.

## Supplementary Information

**Additional file 1.** 

**Additional file 2.** 

**Additional file 3.** 

**Additional file 4.** 

**Additional file 5.** 

**Additional file 6.** 

**Additional file 7.** 

**Additional file 8.** 

**Additional file 9.** 

## Data Availability

Data are available from the authors marine-barjoan.e@chu-nice.fr.
