# Supplementary figures and images for: Impact of social and demographic factors on the spread of the SARS-CoV-2 epidemic in the town of Nice
Source: BMC Public Health. 2023 Jun 6;23:1098. doi: 10.1186/s12889-023-15917-z (PMC10243248; doi:10.1186/s12889-023-15917-z)

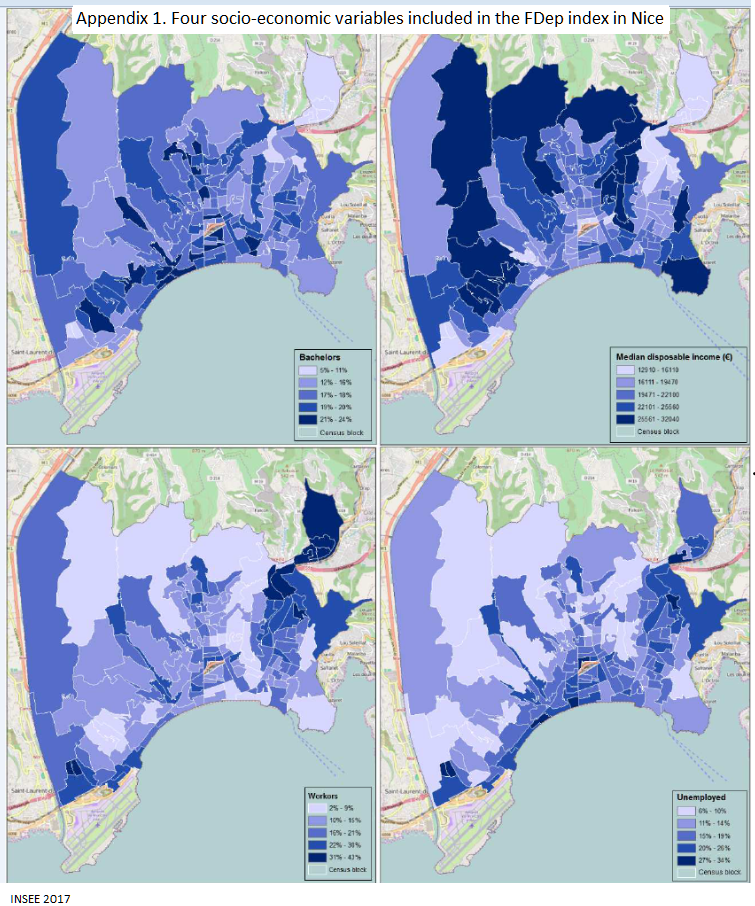

Supplement: Supplementary file 1 — Additional file 1. [file 12889_2023_15917_MOESM1_ESM.tif]

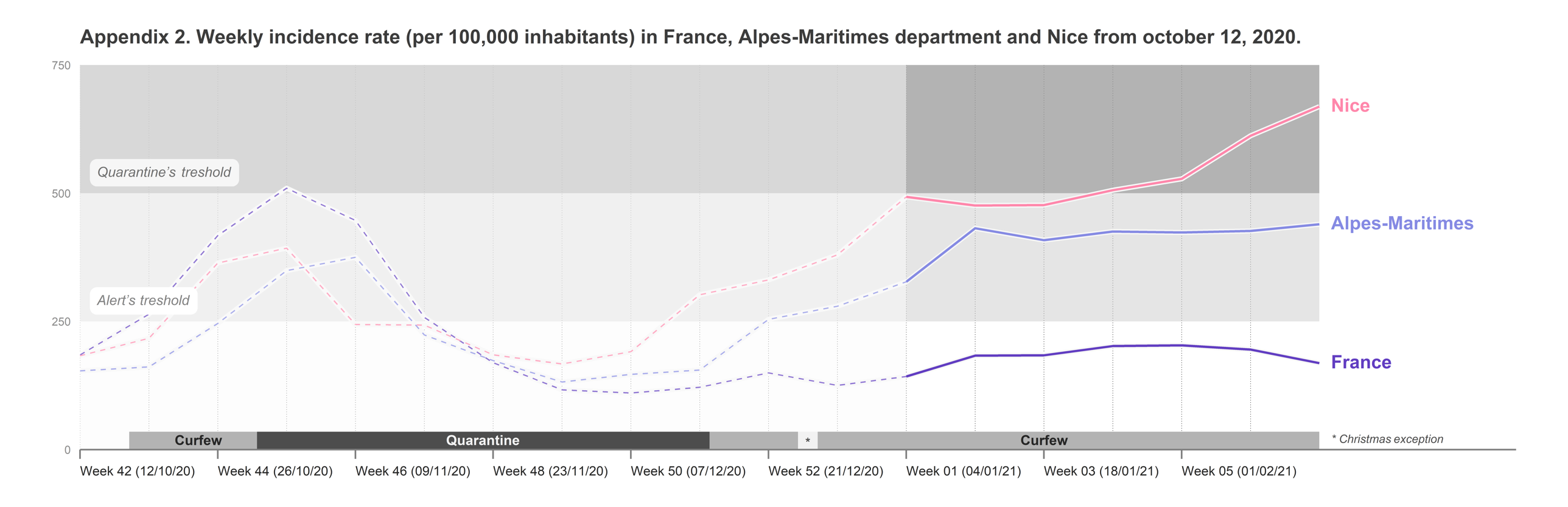

Supplement: Supplementary file 2 — Additional file 2. [file 12889_2023_15917_MOESM2_ESM.tiff]

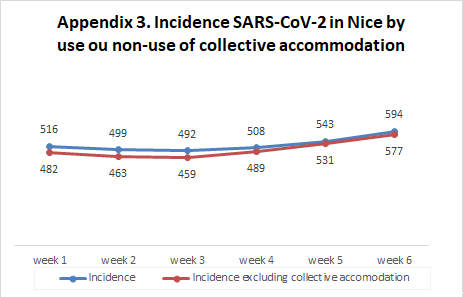

Supplement: Supplementary file 3 — Additional file 3. [file 12889_2023_15917_MOESM3_ESM.tif]

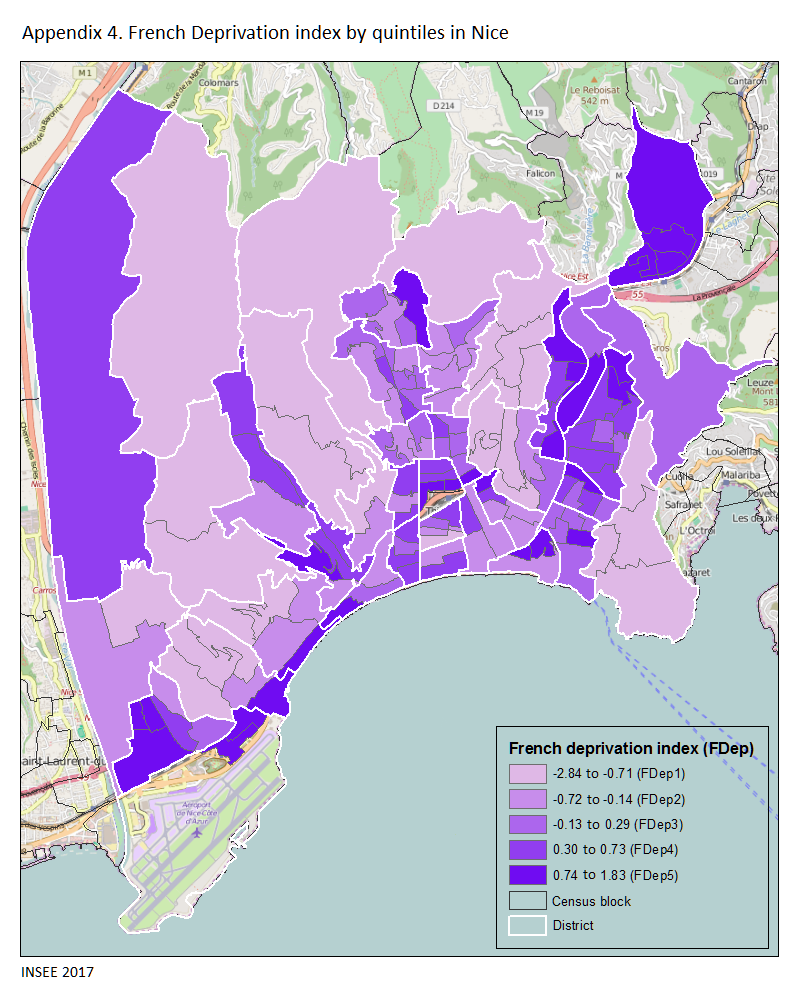

Supplement: Supplementary file 4 — Additional file 4. [file 12889_2023_15917_MOESM4_ESM.tif]

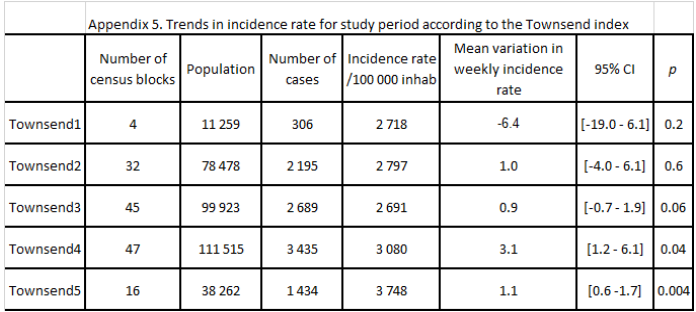

Supplement: Supplementary file 5 — Additional file 5. [file 12889_2023_15917_MOESM5_ESM.png]

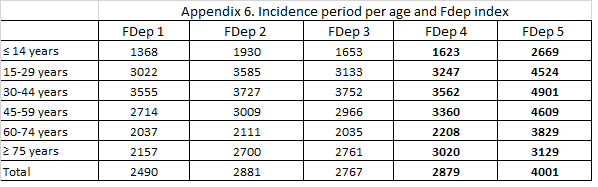

Supplement: Supplementary file 6 — Additional file 6. [file 12889_2023_15917_MOESM6_ESM.png]

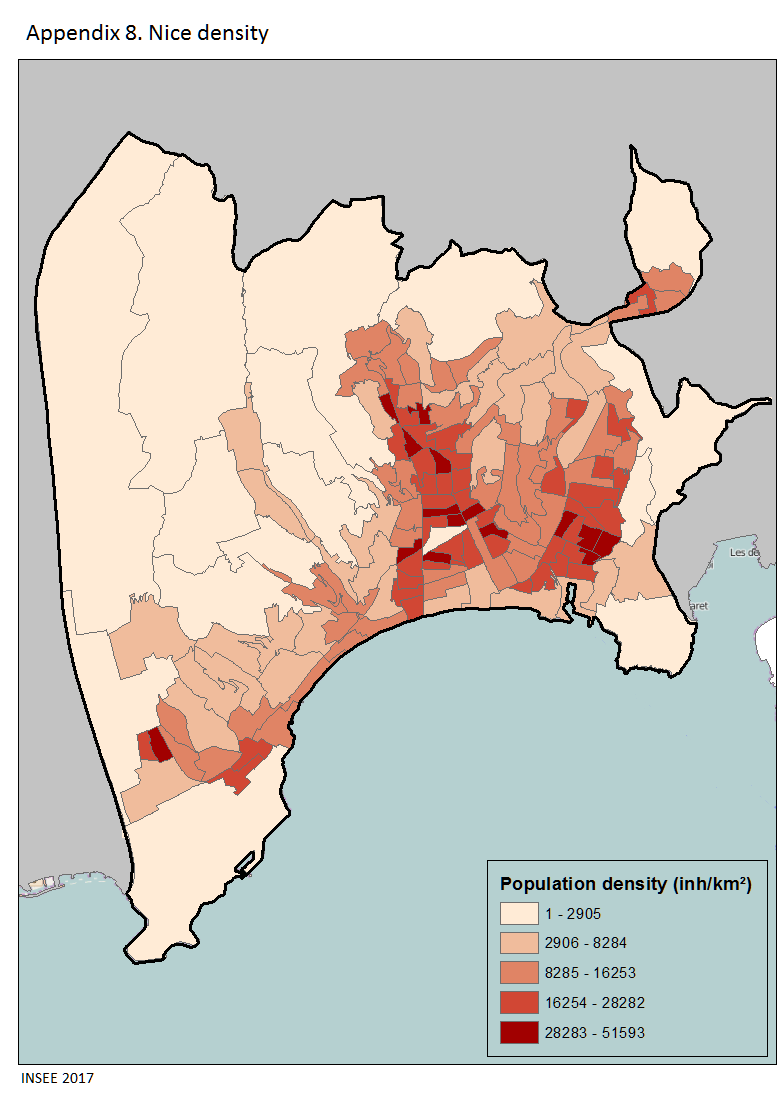

Supplement: Supplementary file 8 — Additional file 8. [file 12889_2023_15917_MOESM8_ESM.tif]

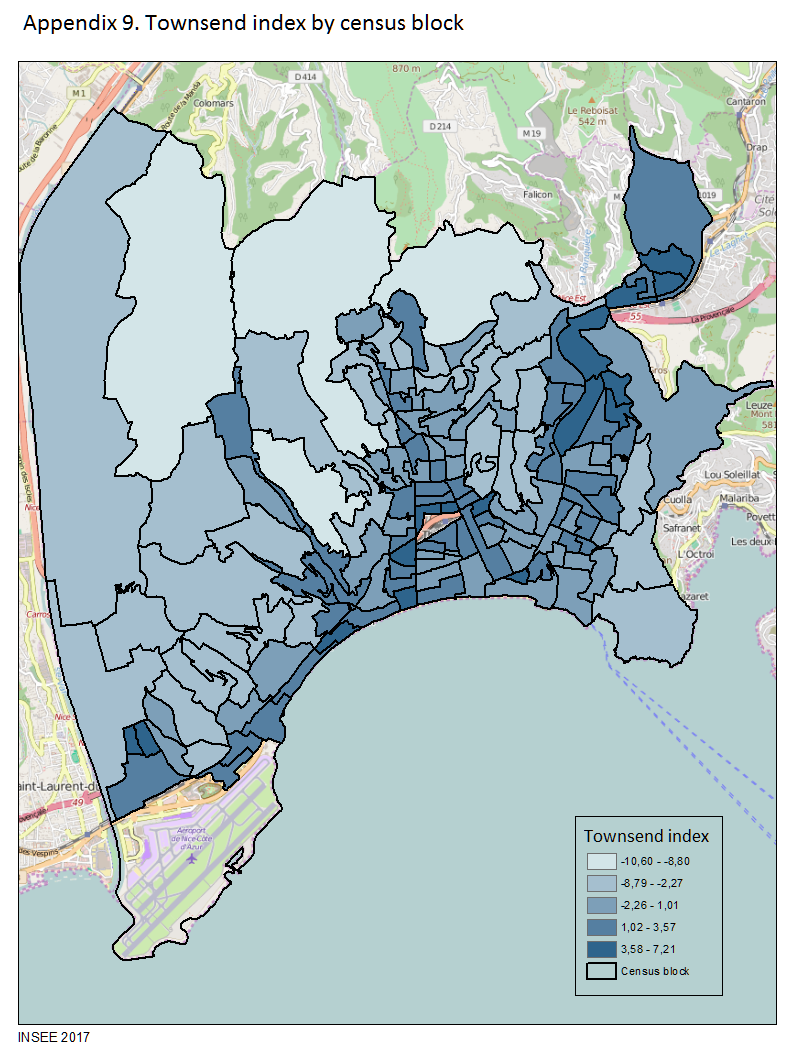

Supplement: Supplementary file 9 — Additional file 9. [file 12889_2023_15917_MOESM9_ESM.tif]
