# Supplementary material for: Impact of social and demographic factors on the spread of the SARS-CoV-2 epidemic in the town of Nice
Source: BMC Public Health. 2023 Jun 6;23:1098. doi: 10.1186/s12889-023-15917-z (PMC10243248; doi:10.1186/s12889-023-15917-z)
Supplement: Supplementary file 7 — Additional file 7. [file 12889_2023_15917_MOESM7_ESM.pdf]

Appendix 7. Socio-economic variables per census blocks

Census block Code	Census block name	Population density	Overcrowded main residences	House as main residence	Appartment as main residence	Owners of main residence	Tenants of main residence	Single- parent families	Poverty rate (%)	Median income (€)	Taxable households (%)	Farmers	Artisans.shopkeepers.company managers	Executives Higher professions	Intermediate professions	Employees	Workers	Unemployed	>14 years with no diploma nor primary school certificate	Population with full state provided health insurance
060880101	SALEYA - CHATEAU	3911	78	9	703	380	323	79	21.3	24430	58.2	0	57	77	116	169	29	76	188	81
060880102	VIEUX NICE - SAINTE REPARATE	25572	134	6	734	212	519	64	31.8	15580	42.6	0	43	93	154	224	97	149	181	
060880103	VIEUX NICE - VISITATION	16806	154	3	1116	351	740	184	30.2	16310	42.1	0	78	172	208	323	148	169	403	142
060880201	GARIBALDI	24443	124	6	1234	605	615	102	17.4	20230	53.8	0	78	279	266	341	112	134	341	153
060880202	PORT	5416	112	0	1057	542	482	130	15.9	21160	54.5	0	73	183	214	223	133	118	268	108
060880203	CASSINI	28282	139	9	1320	520	793	78	19.2	20430	50.3	0	86	189	308	205	124	112	279	154
060880204	BARLA	25245	130	6	1060	415	629	101	18.5	19510	51.3	0	56	186	232	279	144	135	314	136
060880205	RIQUIER - FONTAINE DE LA VILLE	42191	156	0	1174	585	536	101	15.8	20610	54.5	0	82	136	271	351	146	169	357	152
060880206	BONAPARTE	25545	172	17	1417	689	705	184	20.0	20090	52.1	0	83	222	310	368	143	190	444	194
060880301	REPUBLIQUE	29277	193	15	1135	472	672	127	22.0	17100	45.6	0	63	145	237	418	189	180	346	264
060880302	PIERRE SOLA	20487	206	6	1104	435	649	118	26.5	16780	42.5	3	40	116	222	369	216	185	458	211
060880303	TNL - BEAUMONT	23283	147	3	1063	515	536	116	15.4	20350	54.4	3	54	178	234	274	91	135	292	107
060880304	RIQUIER - ARSON	29753	245	11	1665	701	947	191	19.6	18590	51.0	0	90	198	363	489	241	293	552	262
060880305	RIQUIER	36283	219	20	2054	884	1150	203	19.0	19340	50.1	0	133	241	494	672	273	216	660	225
060880401	MONT BORON	1286	53	136	842	689	267	55	11.0	35730	72.9	0	85	220	125	114	22	73	273	27
060880402	CARNOT	6011	67	36	809	378	429	62		25090	59.8	0	65	132	158	163	76	90	246	28
060880403	MONT ALBAN	3653	80	112	1133	874	352	97	9.0	26870	66.4	0	107	273	336	331	123	112	278	58
060880404	BISCHOFFSHEIM	2647	38	225	452	514	139	39		30670	70.4	3	81	211	101	132	22	67	114	25
060880501	DIABLES BLEUS	23516	288	45	2016	773	1232	166	20.3	18470	47.5	0	98	196	412	788	359	331	831	344
060880502	SAINT JEAN D'ANGELY	17903	313	19	1895	617	1367	207	21.4	17990	47.4	0	70	172	463	616	313	355	699	318
060880503	SAINT ROCH - JEAN XXIII	25244	147	67	1102	650	514	129	14.8	20430	54.3	0	47	127	280	385	140	134	381	144
060880504	VAUBAN	16253	197	0	1518	576	877	208	15.1	18760	49.2	0	70	107	400	634	318	268	628	262
060880505	SAINT ROCH - RICOLFI	21252	139	8	1117	509	588	108	15.9	19660	48.7	0	36	86	188	529	162	146	461	118
060880506	SAINT CHARLES	14913	138	83	1102	389	766	174	20.6	16700	38.7	0	47	55	178	532	213	232	816	274
060880507	ROQUEBILLIERE	11981	186	4	1408	416	990	199	19.0	17370	42.6	0	47	93	238	585	301	202	679	
060880508	PIERRE SEMARD	17915	307	9	1108	85	1038	235	38.3	10590	22.2	3	29	9	99	454	375	261	753	
060880509	MONT GROS	11645	190	17	578	0	595	179	44.5	8170	18.3	0	24	10	31	293	172	196	492	
060880601	LYAUTEY - GENDARMERIE	22803	178	6	977	494	477	153	26.7	15310	38.1	1	49	46	231	367	276	160	908	491
060880602	PASCHETTA	11057	108	75	689	223	542	123	22.7	17220	42.1	0	44	53	114	236	188	90	291	

060880603	PASTEUR	15100	218	28	1304	495	806	187	23.8	16660	40.5	0	41	43	213	481	239	164	747	324
060880604	PASTEUR - VOIE ROMAINE	25305	243	0	1262	393	856	124	29.9	13590	33.9	0	65	36	123	404	280	137	617	
060880605	PASTEUR - SAINT PONS	5230	145	35	555	209	352	96	36.7	11250	25.0	0	38	29	110	175	250	111	508	
060880701	ROUTE DE TURIN	7198	276	3	1135	96	1044	211	39.2	10360	26.6	0	35	35	224	430	252	232	670	
060880702	BON-VOYAGE	7597	394	64	1252	320	990	287	44.9	8840	20.8	0	32	55	212	637	496	297	944	
060880801	VINAIGRIER	693	89	320	319	353	267	94	26.3	20280	52.8	0	39	126	84	225	133	118	250	306
060880901	ARIANE - LES CHENES	15583	217	0	901	9	892	229	44.8	8380	17.6	0	54	0	48	307	319	217	531	
060880902	ARIANE - MONZIE	18414	199	0	757	413	323	59	37.6	11400	26.2	0	152	6	84	170	208	87	379	
060880903	LA LAUVETTE	893	137	95	439	96	432	103		10930		0	61	4	48	128	134	56	376	
060880904	ARIANE - RIPERT	14239	172	21	587	119	490	90	48.7	8610	17.5	0	64	1	44	126	180	85	312	704
060880905	ARIANE - SARAMITO	9875	315	10	781	186	600	115	45.9	9450	19.9	0	102	9	57	189	262	127	399	
060881001	CIMIEZ - MONASTERE	5618	79	138	907	726	299	91		27890	67.4	0	65	270	190	192	105	92	232	41
060881002	BRANCOLAR - REGINA	9150	148	128	991	628	501	89	10.2	29100	68.2	0	102	329	251	271	121	199	280	54
060881003	BRANCOLAR - SCUDERI	5905	66	145	684	597	204	64		25470	64.8	0	54	213	240	198	45	72	151	74
060881004	CAP DE CROIX	5593	62	100	1095	861	301	143	8.4	29470	69.8	0	54	319	289	285	73	99	308	50
060881005	RIMIEZ	3381	107	214	1300	1039	394	147	8.2	28850	70.4	6	84	383	339	336	67	102	266	62
060881101	PUGET	20072	207	85	1610	864	760	201	15.2	21330	53.1	0	75	308	453	442	142	242	388	209
060881102	PARC CHAMBRUN	11478	202	152	1440	771	749	144	12.9	24090	58.5	0	109	331	466	375	173	164	311	164
060881103	LE RAY - GRAVIER - DUNANT	8284	119	124	1103	906	285	130	12.0	22700	60.1	0	71	258	377	300	129	68	213	120
060881104	GRAVIER	11733	91	12	944	589	366	104	12.6	22590	57.5	0	55	122	194	311	119	91	321	77
060881105	HENRY DUNANT	10536	96	39	1020	467	556	123	18.4	18850	47.5	0	59	176	202	324	170	172	324	198
060881201	NICE-NORD - SAQUI	7216	152	38	1238	529	731	208	26.2	15460	40.3	0	36	87	207	428	260	204	654	357
060881202	COMTE DE FALICON	12059	50	11	645	520	119	86		20560	51.2	3	28	72	133	241	89	55	241	73
060881203	CERNUSCHI	8714	95	162	865	707	289	94	17.1	21690	58.0	0	81	163	293	278	109	109	253	115
060881204	LAS PLANAS - LA VALLIERE	9159	176	181	1304	719	744	204		19130		0	79	158	361	558	265	229	657	
060881301	CESSOLE	33583	186	3	1172	544	609	144	20.0	18000	45.8	0	48	110	270	429	200	207	418	210
060881302	SAINT BARTHELEMY	21670	170	33	1276	699	671	138	16.4	20480	50.9	0	71	211	231	362	145	98	404	113
060881303	GORBELLA	36859	214	26	1265	689	584	125	13.9	20780	53.1	0	80	142	266	394	190	107	321	116
060881304	SAINT SYLVESTRE	15732	108	63	1037	527	529	113	10.4	21620	56.3	0	58	84	241	353	212	116	373	80
060881305	VILLA ARSON	18573	229	77	1245	633	656	116	16.5	20400	51.8	0	77	136	235	443	212	163	357	146
060881306	CYRNOS	16563	99	174	779	638	304	62	11.9	22730	56.9	0	42	166	225	219	105	55	339	76
060881307	CYRILLE BESSET - LA FORET	8041	109	170	963	715	391	106	11.1	23470	61.8	0	109	185	346	343	152	122	376	73
060881308	LA CLUA	9143	82	115	970	582	476	116	15.4	20640	51.7	0	67	137	273	386	126	77	241	73
060881309	CANAVESE	39307	171	8	1354	726	594	126	18.3	18400	47.6	0	62	121	302	405	175	168	533	204
060881401	EVECHE	17757	199	79	1153	569	632	113	15.5	20740	52.1	0	51	160	247	418	201	191	393	155

060881402	BELLEVUE	22453	132	46	1075	565	498	126	19.4	21200	51.8	0	88	228	246	355	123	188	331	164
060881403	LE RIGHI	14722	137	54	1117	660	474	82	18.5	21830	54.9	0	52	191	228	283	110	111	324	161
060881404	MANTEGA	9684	99	207	625	340	494	108	18.8	18750	47.5	0	43	143	176	225	155	90	267	136
060881501	MARCEAU	35127	162	3	928	418	504	124	27.9	15950	42.1	0	51	150	185	286	168	142	356	
060881502	THIOLE	27503	156	7	1173	532	608	100	18.2	20030	51.8	0	67	209	231	360	146	152	362	154
060881503	JEANNE D'ARC - FUON-CAUDA	21059	80	0	1049	489	513	86	15.9	21310	54.8	0	47	258	281	242	60	118	310	148
060881504	MICHEL ANGE	23127	124	22	991	387	589	93	16.1	20690	53.8	0	34	229	241	245	53	115	245	139
060881505	MICHELET	30714	185	21	1208	453	731	136	22.2	17140	46.7	0	61	175	286	347	99	94	360	182
060881506	BORRIGLIONE - SAINT LAMBERT	20750	127	52	1114	532	617	103	18.7	20900	55.1	0	49	219	267	302	120	134	309	108
060881601	CARABACEL	9140	82	41	982	630	343	109	12.2	29600	66.6	0	85	282	228	238	67	142	207	80
060881602	ACROPOLIS - XVE CORPS	9844	159	39	1073	524	555	146	21.0	18820	47.8	0	63	147	324	447	179	127	477	194
060881603	SAINTE ROSALIE - ARBRE INFERIEUR	8680	156	168	1326	808	650	208	13.3	23270	62.1	6	69	301	355	368	198	138	280	167
060881604	CIMIEZ	9237	115	61	1414	1024	408	121	9.5	35700	75.4	0	114	584	321	204	27	125	136	49
060881605	CARAVADOSSI - GEORGE V	8645	78	31	842	548	308	75	13.5	25190	62.5	0	87	231	201	150	34	87	254	97
060881606	CIMIEZ - VALROSE	5845	70	76	1020	756	323	90	9.1	31110	72.7	0	70	379	248	183	37	68	204	46
060881701	PROMENADE DU PAILLON	10962	82	0	852	305	522	138	18.1	24560	60.7	3	86	216	206	200	44	127	183	91
060881702	HOPITAL SAINT ROCH	15977	72	0	1026	391	625	75	16.5	23100	54.8	0	76	289	244	265	69	112	134	76
060881703	RAIMBALDI	24446	160	3	927	273	640	132	29.4	14260	36.8	0	23	104	160	309	179	197	364	
060881704	DESAMBROIS	38613	161	0	1126	421	700	214	24.3	18830	50.1	0	76	184	329	438	163	219	344	169
060881705	SASSERNO	21651	98	3	1090	485	611	101	19.3	21790	52.6	1	61	260	218	222	110	157	363	130
060881802	VERNIER	27415	254	24	1106	484	628	134	35.5	12830	34.2	0	50	105	176	434	236	335	521	
060881803	TRACHEL	51593	207	6	1042	310	725	123	35.6	12620	31.7	0	83	90	208	312	219	205	435	
060881804	CLEMENT ROASSAL	34885	189	6	1354	539	774	133	24.7	16650	44.1	0	84	203	318	354	223	190	518	370
060881805	GARNIER - GARE DE PROVENCE	19101	123	6	1061	439	585	111	22.6	18780	47.4	0	54	177	240	314	160	178	380	
060881901	GROSSO	32666	203	18	1152	532	619	152	26.0	16300	41.0	0	69	167	240	345	179	172	400	274
060881902	EGLISE RUSSE	14251	130	17	986	513	447	43	18.2	21800	53.2	0	51	165	255	237	111	140	216	160
060881903	PARC IMPERIAL	12541	155	78	1056	549	542	97	24.3	18800	45.7	0	58	215	250	266	138	176	368	219
060882001	LE PIOL - CYRNOS	4329	67	144	799	697	214	75	12.3	26910	66.8	0	47	166	240	182	107	86	278	81
060882002	SAINT PHILIPPE	5625	137	104	860	427	470	111	15.4	21790	57.9	0	64	184	327	291	101	159	222	112
060882101	CARLONE	13654	162	41	1157	584	588	95	18.3	21290	54.7	0	77	197	283	342	142	197	263	99
060882102	MADELEINE - ROBIONI	13079	252	223	1180	532	838	189	23.4	16400	40.9	5	65	113	325	525	287	241	546	
060882103	LA BORNALA	8684	168	73	1218	325	949	148	31.7	14180	35.3	0	58	124	234	479	239	245	351	275
060882104	MADELEINE	7996	144	165	634	350	477	94	24.1	17890	46.3	0	29	52	133	258	145	147	339	
060882201	LIBERTI - ALBERT 1ER	4793	64	3	752	255	464	33	20.4	23010	56.3	0	80	128	111	116	39	58	175	

060882202	NOTRE-DAME	18036	177	1	1241	413	833	139	29.8	17510	48.0	0	76	252	254	323	166	223	267	
060882203	JEAN MEDECIN	10504	201	3	1581	680	954	131	20.0	21170	55.8	0	107	303	299	301	184	210	320	
060882301	MUSICIENS	12141	43	14	693	366	323	41	18.2	26070	59.4	0	41	115	101	119	78	91	152	110
060882302	ROSSINI	25055	107	9	928	491	417	57	19.1	22180	55.0	0	94	183	113	145	62	95	182	116
060882303	CLEMENCEAU	32853	180	3	1174	578	546	91	23.1	19520	53.4	0	79	210	243	267	87	163	271	172
060882304	THIERS - DURANTE	22982	173	0	1298	521	748	97	22.9	18220	48.4	0	55	234	253	422	143	202	410	
060882401	FRANCE - NEGRESCO	8226	119	4	938	393	499	59	31.7	17530	46.6	0	92	124	127	197	59	128	245	
060882402	VICTOR HUGO - BUFFA	12850	92	10	1172	583	540	86	22.3	22200	55.4	0	81	220	147	181	44	118	316	146
060882501	DANTE	17861	152	7	1033	317	714	76	31.0	16980	43.8	0	67	145	161	218	94	220	278	157
060882502	BOTTERO	20920	195	9	1181	407	750	96	24.3	18390	46.8	0	116	120	198	267	145	187	338	185
060882503	POTIERS	23705	168	21	1027	506	530	53	24.5	18900	49.3	0	56	140	135	239	161	171	251	
060882504	CHATEAUNEUF	24686	274	8	1525	577	896	190	28.3	16550	45.3	0	93	163	263	371	228	279	478	
060882601	LENVAL	13950	212	0	971	378	568	98	35.2	14100	36.4	0	62	90	118	274	120	208	230	228
060882602	MAGNAN	11551	181	4	1088	405	662	113	25.7	19330	47.5	0	64	163	219	293	90	201	215	
060882603	BAUMETTES	8216	135	60	974	528	488	73	18.0	21660	54.9	3	85	127	204	256	98	131	318	128
060882701	LA CALIFORNIE	11481	227	13	1262	514	743	78	29.0	16220	41.1	0	79	101	208	306	169	180	369	245
060882702	PASTORELLE - BOSQUETS	4572	157	212	895	682	365	104	14.7	22630	58.0	0	69	223	245	276	125	150	324	117
060882703	FACULTE DE LETTRES	6643	39	122	607	475	228	33	18.7	22430	55.3	0	64	124	176	151	36	96	105	83
060882704	GATTAMUA	6510	128	33	1488	1032	438	122	9.3	27910	71.4	0	123	338	480	389	91	184	232	67
060882705	ABBAYE DE ROSELAND	5733	85	81	1109	722	414	87	11.5	29980	69.3	0	114	319	237	195	102	85	177	37
060882801	CARRAS	11240	301	90	1585	784	859	137	28.4	15860	39.5	0	93	123	254	418	303	243	650	391
060882802	CAUCADE	6629	184	95	1845	1178	689	200	9.4	24940	63.5	6	111	365	558	663	229	222	351	83
060882803	LANTERNE - MANDARINIERS	11423	91	70	908	519	438	130	11.2	21710	57.5	0	54	164	300	292	129	168	307	109
060882804	LANTERNE	7194	140	99	1424	990	496	121	9.8	28230	70.7	0	177	313	395	351	93	122	213	47
060882805	NAPOLEON III	5924	110	47	1447	983	490	117	8.5	29500	71.3	0	128	392	290	337	78	95	232	54
060882901	LES SIAGNES	3227	179	5	685	345	324	104	42.0	11300	22.9	0	53	44	96	305	224	173	784	
060882902	LES MOULINS	38192	351	7	1395	29	1383	416	46.8	8730	17.5	0	54	29	152	725	611	564	1560	
060882903	DIGUE DES FRANCAIS	28082	181	0	858	0	867	235		8720		0	41	8	83	322	346	271	919	
060882904	PAUL MONTEL	10127	156	20	789	438	363	147	19.7	17520	43.8	0	48	44	166	366	260	155	477	180
060882905	LA VICTORINE - GRINDA	8834	84	50	879	571	332	110	15.1	20320	51.7	0	70	102	244	358	155	93	358	115
060882906	PLATEAUX FLEURIS	12890	198	18	1425	822	591	139	10.2	22760	61.1	1	69	188	427	472	243	167	261	90
060883002	ARENAS - CASSIN	16538	237	9	1134	665	455	148	31.3	13940	34.5	0	79	98	219	425	313	255	609	354
060883003	SAINT AUGUSTIN	17479	224	28	1386	658	739	140	32.7	14970	37.0	0	38	88	232	452	335	188	677	296
060883101	SPAGNOL - SAINTE MARGUERITE	3636	130	231	1351	937	627	132	8.7	27650	70.0	0	136	483	576	489	206	195	291	62
060883102	PARC DES SPORTS	1523	161	72	1450	736	771	192	9.7	23390	62.1	10	114	326	577	561	295	186	401	146

060883201	FABRON - TERRON - ARCHET	1595	114	377	892	871	336	117	9.9	27720	69.6	6	140	319	404	349	204	165	502	54
060883202	SAINT ANTOINE GINESTIERE	1333	68	518	514	792	215	90		30220	72.4	4	121	326	387	277	119	127	395	32
060883301	LINGOSTIERE - SAINT ISIDORE	401	146	608	701	588	640	197	31.1	17640	38.1	17	174	191	401	478	277	178	773	228
060883401	BELLET - MAGNAN	2615	96	303	720	620	367	103	14.3	22140	56.8	0	79	206	387	350	164	116	343	139
060883402	MADELEINE - NICOLAI	5146	161	86	864	493	419	145	16.8	18480	47.2	0	65	112	234	384	177	127	357	
060883403	MADELEINE SUPERIEURE	3221	146	146	715	327	518	114	20.9	17040	41.7	0	53	59	157	324	193	164	515	181
060883501	CREMAT - BELLET	329	45	777	123	753	102	52		29160	69.8	22	139	278	334	245	98	106	269	38
060883601	SAINT PIERRE DE FERIC	1385	83	397	820	714	489	152	8.7	26290	66.6	15	81	201	371	473	204	199	283	63
060883602	COSTIERE	1857	77	246	810	900	127	97	9.4	25600	65.6	0	117	192	430	374	134	100	289	53
060883701	SAINT PANCRACE - PESSICART	671	62	1075	466	1250	252	119	7.4	28770	70.7	10	207	452	407	429	120	*150*	350	87
060883801	GAIRAUT	993	26	855	304	917	218	106		34180	76.0	6	113	327	312	255	79	*118*	367	26
